# Reduction of G_0_ phase cells of colon cancer caco-2 cells may enhance 5-fluorouracil efficacy^☆^

**DOI:** 10.1016/S1674-8301(10)60010-3

**Published:** 2010-01

**Authors:** Lin Ye, Kaixiong Tao, Yang Yu, Guobin Wang

**Affiliations:** Department of Laproscope Surgery, Union Hospital affiliated to HuaZhong University of Science and Technology, WuHan 430022, China

**Keywords:** epidermal growth factor, chemosensitivity, induction, dormant cells

## Abstract

**Objective:**

A major problem in the chemotherapy of colon caner may be due to those cells that are in residence in the G_0_ phase where they are less vulnerable to conventional therapy. To overcome this phenomenon, we attempted to recruit the reentry of these cells into the cell cycle via a signaling pathway that manipulates tumor growth.

**Methods:**

Epidermal growth factor (EGF) was used to stimulate colon cancer caco-2 cells. FACS analysis and proliferating cell nuclear antigen (PCNA) staining were used to estimate the cell cycle transition and cell proliferation activated by EGF, and a MTT assay was used to evaluate the synergistic effect of EGF and chemotherapy.

**Results:**

The percentage of caco-2 cells in the G_0_/G_1_ phase was significantly reduced by nearly 20% and the percentages in the S and G_2_/M phases were increased by EGF. The combined use of EGF and 5-fluorouracil (5-FU) enhanced the caco-2 cell chemosensitivity to 5-FU, reaching a maximum of an approximately threefold greater sensitivity than to 5-FU alone as judged by the 50% inhibiting concentration (IC_50_).

**Conclusion:**

Our study demonstrated that stimulation by EGF enhanced the chemosensitivity of caco-2 cells to 5-FU, which may be a novel therapeutic protocol in colon cancer.

## INTRODUCTION

Colon cancer is still a leading cause of cancer-related mortality in the world. Although its incidence and mortality have decreased[1] due to remarkable improvements in surgical technique, radiation therapy, and systemic therapies[2], chemoresistance is still a hurdle in the treatment of colon cancer. The lack of responsiveness to chemotherapy[3] is one of the most important problems that needs to be resolved.

The chemotherapy unresponsiveness of colon cancer cells is most likely due to a multiplicity of causes[4]. One plausible reason is the large number of cells in the G_0_ phase, cells that are less vulnerable to cell cycle dependent chemotherapy[5]. Furthermore, these cells may be activated anytime, anywhere by growth factors[6] or cytokines[7],[8], and they may be a time bomb for tumor recurrence. This poses a hurdle in the goal of modern cancer treatment where the goal is the prolongation of disease free survival. The aim of this study is to reduce the number of cells in the G_0_ phase and thus enhance the chemotherapy efficacy in a colon cancer model.

Epidermal growth factor receptor (EGFR) was reported to be overexpressed in colon cancer[9]. The activation of this receptor plays an important role in the regulation of tumor growth[10]. In this study, we use epidermal growth factor (EGF), a key trigger in activating the EGFR signaling pathway[11]–[13], to stimulate caco-2 colon carcinoma cells which were tested to overexpress EGFR. We found that the percentage of tumor cells in G_0_ phase was reduced and more tumor cells were synchronized in the S phase and G_2_/M phase. Meanwhile, in the presence of EGF, the chemosensitivity was enhanced nearly threefold. This result may provide a novel strategy for future therapy.

## MATERIALS AND METHODS

### Cell culture

Cells from the caco-2 human colon cancer cell line were purchased from the China Center for Type Culture Collection, and were grown in High Glucose DMEM medium (Hyclone, USA) containing 10% fetal bovine serum (Gibco, USA) with penicillin and streptomycin antibiotics (100 U/ml penicillin, 100 µg/ml streptomycin, respectively) at 37°C in a humidified incubator with 5% CO_2_ in air.

### Cell cycle analysis

Tumor cells (2×10^5^) were seeded in 6-well plates. After overnight incubation, the medium was changed with serum-free medium containing various amounts of EGF (PeproTech, USA) and incubated for 48h. Cells were then harvested and stained with propidium iodide for DNA cell cycle analysis using standard FACS techniques and FACSort (BD, USA) The results were analyzed and expressed as percentages of total gated cells using the Modfit LT^TM^ Software (BD, USA).

### Western blot analysis

Briefly[14], whole cell extracts (50 µg/lane) were electrophoresed through 10% sodium dodecyl sulfate (SDS)-polyacrylamide gel and were transferred onto a Hybond-C-super nitrocellulose membrane (Dako, Denmark). Prestained molecular weight markers (Dako) were included. Membranes were blocked for 1h in Tris-buffered saline (TBS, pH 7.5) with 0.5% Tween-20 (TBST) and 5% nonfat dry milk. After blocking, membranes were incubated for 2 h with proliferating cell muclear antigen (PCNA) (1:100 dilution, Wuhan Boster, China). After incubation with horseradish peroxidase conjugated secondary antibody (1:5000 dilution, Boster), the membranes were scored by the enhanced chemiluminescence (ECL) detection system (Amersham, USA).

### MTT assay

Cells in 100 µl of culture medium per well were seeded into 96-well plates (Dakewe, Shenzhen, China) and cultured at 37°C for 24 h. Then the culture medium was replaced with serum-free medium and 10 µl of medium containing various amounts of 5-fluorouracil (5-FU) (HaoranBio, China) and EGF (PeproTech, USA) prepared using serum free medium, was added to each well. After additional incubation at 37°C for 48 h, 10 µl of MTT (Boster) dissolved in PBS at a concentration of 5 mg/ml was added to each well, and the plates were incubated at 37°C for 4 h. The medium was removed, 150 µl of dimethylsulfoxide was added to each well, and the plates were agitated for 5 min. The absorbance was then read at 570 nm in a scanning spectrophotometer.

### Statistical analysis

All data are expressed as mean ±SD. Differences among groups were analyzed by one-way analysis of variance (ANOVA), and Fisher's Least Significant Difference (LSD) method was used for multiple comparison. The P-value reported was two-sided and a value of *P* < 0.05 was considered statistically significant. All analyses were performed using the SPSS software (Version 11.0, SPSS Inc., USA).

## Results

### Cell cycle transition by stimulation with EGF

**Fig. 1:**
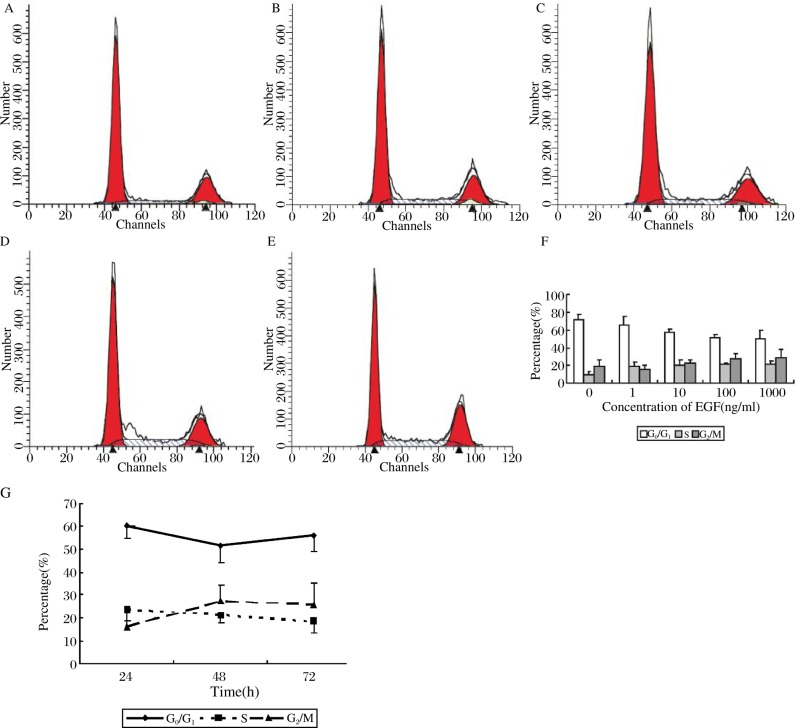
Effect of EGF on cell cycle transitioning. A-E: Cell cycle analysis with different concentrations of EGF (0-1 000 ng/ml). F: The cell cycle distribution correlated with the concentration of EGF, and the most evident transition was at a concentration of 100 ng/ml where the proportion of cells in the G_0_/G_1_ phase was reduced by nearly 20% compared to the control group (*P* < 0.05). *F* = 9.055, *P* = 0.002, within subject effect *P*_1-2_ = 0.191, *P*_1-3_ = 0.007, *P*_1-4_ = 0.001, *P*_1-5_ = 0.001, *P*_4-5_ = 0.765 and 1 to 5 represent the EGF concentrations of 0 to 1 000 ng/ml respectively. G: Effect of EGF (100 ng/ml) on cell cycle transitions at different time point (24 h, 48 h, 72 h). The percentage of G_0_/G_1_ phase cells was reduced significantly in 48 h by nearly 10% (Effect of time *F* = 5.774, *P* = 0.028).

We stimulated tumor cells with EGF of different concentrations. As shown in ***Fig. 1A-1F***, the cell cycle transitions were EGF concentration dependent at concentrations at or below 100 ng/ml. At the concentration of 100 ng/ml, the percentage of cells in the G_0_/G_1_ phase was reduced by approximately 20% compared to the control group (*P* < 0.05), and the percentage of cells in the S and G_2_/M phases increased. The percentage of cells transitioning out of the G_0_/G_1_ phase did not significantly change further when the concentration of EGF was above 100 ng/ml. Then we stimulated tumor cells with an EGF co ncentration of 100 ng/ml for different time periods (***Fig. 1G***), and found that the number of G_0_/G_1_ phase cells was reduced significantly at 48 h by 10% compared to 24 h (*P* < 0.05).

### Expression of PCNA following stimulation with EGF

**Fig. 2:**
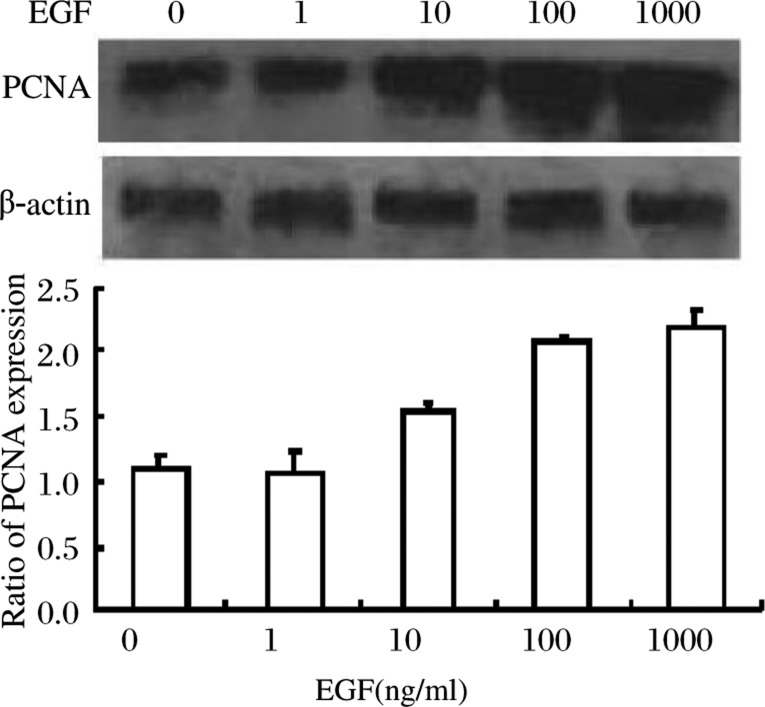
Effect of EGF on the expression of PCNA. The expression of PNCA was EGF concentration dependent (0∼1 000 ng/ml), reaching a maximum level of > 2 times that of the control group (*P* < 0.05). *F* = 83.733, *P* < 0.001, within subject effects *P*_1-2_ = 0.692, *P*_1-3_ < 0.001, *P*_1-4_ < 0.001, *P*_1-5_ < 0.001, *P*_4-5_ = 0.134, and 1 to 5 represent the EGF concentrations of 0 to 1 000 ng/ml respectively.

PCNA correlates with the proliferation of cells in many human tumors, including colon cancer. Levels increase in late G_1_ phase and peak in the S phase of the cell cycle, and the antigen is not detectable in quiescent cells. In our experiment, the expression of PCNA increased with increasing concentrations of EGF and the maximum increase was > 2 fold (***Fig. 2***) when the EGF concentration was≥100 ng/ml. This demonstrated the dormant cells (G_0_ phase) were reduced and were recruited into an activated phase.

### Chemosensitivity enhanced by stimulation with EGF

**Fig. 3:**
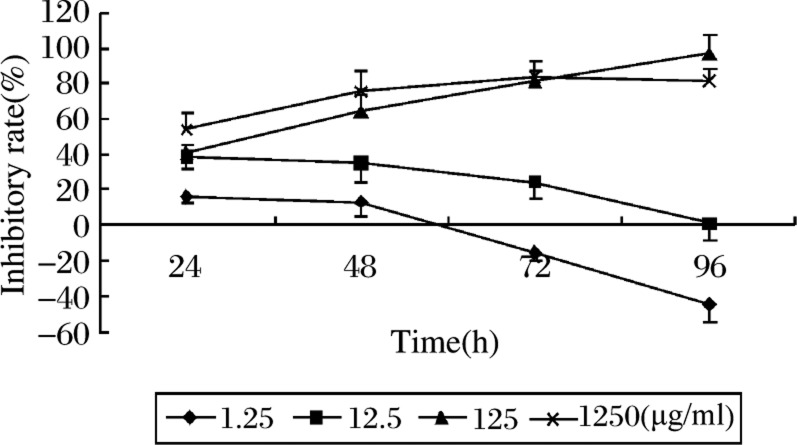
Effect of 5-FU on growth of caco-2 cells. Cells were seeded in 96-well microplates and incubated in DMEM medium over night. Then various concentrations of 5-FU (1.25∼1250 µg/ml) were added. After further incubation for different time periods (24∼96h), cell viability was evaluated by MTT assay. Means±SD of triplicate experiments. When incubated for more than 72 h, a 5-FU concentration of less than 12.5 µg/ml did not inhibit the tumor cell growth, and at the incubation of 24 h, the concentrations of 5-FU higher than 125 µg/ml did not achieve the maximal effect. (Effect of time *F* = 40.127, *P* < 0.001; effect of 5-FU levels: *F* = 3767.636, *P* < 0.001).

We evaluated the synergistic effect of EGF and 5-FU using an MTT assay. The relative sensitivity was judged by the 50% inhibiting concentration (IC_50_) . The growth of caco-2 cells was inhibited in a concentration-dependent manner by 5-FU over the concentration range 1.25 to 1 250 µg/ml (***Fig. 3***). The sensitivity of caco-2 cells to 5-FU was significantly enhanced by a combination with EGF, and this sensitivity effect was increased nearly threefold compared to the group that was treated with 5-FU alone (***Table 1***).

**Table 1:** Combined effect of 5-FU and EGF on growth of caco-2 cells.

Treatment	IC_50_ (µg/ml)	Ratio*
5-FU	54.11	
+EGF (1ng/ml)	39.58	1.37
(10ng/ml)	25.40	2.13
(100ng/ml)	18.75	2.89
(1 000ng/ml)	20.41	2.65

*Relative sensitivity compared to 5-FU alone.

### Effect of 5-FU and the synergistic use of EGF on cell cycle transition

**Fig. 4:**
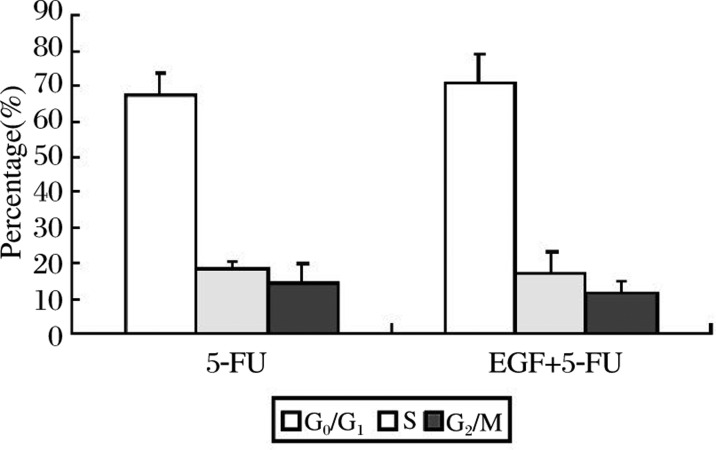
Effect of 5-FU (1 250 ug/ml) on cell cycle transition. Although the cells in G_0_/G_1_ phase increased nearly 5% when treated with 5-FU (1 250 µg/ml) plus EGF (100 ng/ml) compared to when treated with 5-FU alone, there was no significant difference in cell cycle distribution (*P* > 0.05).

As shown in ***Fig. 4***, although the cells in the G_0_/G_1_ phase increased nearly 5% when treated with 5-FU (1 250 µg/ml) plus EGF (100 ng/ml) compared to when treated with the 5-FU alone, there was no significant difference on cell cycle transition (*P* > 0.05).

## DISCUSSION

Chemo-resistance and recurrence become the major problems in the treatment of colon cancer. Conventional therapies which target actively dividing cells may substantially reduce tumor bulk, but often times do not prevent tumor regrowth, presumably because conventional therapy does not destroy the G_0_ cells which are in a dormant state[15]. Thus the long-term effect of chemotherapy may be poor as any activation of G_0_ phase cells may result in recurrence.

There are few studies dealing with the G_0_ phase cells. Some investigators reported that the unresponsiveness of leukemic cells to chemotherapy could be due to their residence in the resting G_0_ phase of the cell cycle[3],[5], and recruitment of leukemic cells from the dormant phase into an activated phase of the cycle by activation or induction of proliferation restored their sensitivity. Hambek and coworkers[6] found that the toxicity of docetaxel in head and neck cancer treatment could be enhanced by stimulation of G_0_ cells which were resistant to chemotherapy.

In our study, we found that the number of G_0_ phase cells was reduced and more tumor cells were recruited into an activated phase by EGF, while the toxicity of 5-FU was enhanced nearly threefold. These results support our contention that caco-2 cells become more vulnerable to chemotherapy when there is a reduction of dormant cells by stimulation of EGF. The same result was found in another colon cancer cell line (sw480). With this cell line the 5-FU chemosensitivity was nearly doubled by the synergistic use of 5-FU with EGF compared to the use of 5-FU alone (data not shown).

To date, many studies on the treatment of colon cancer mainly target signaling molecules which manipulate the key signaling pathways regulating tumor growth[16]–[19]. Although clinically meaningful antitumor effects were observed in patients with advanced or metastatic colon cancer in some clinical trials[20],[21], the dormant cells (G_0_ phase) were still ignored. As a result, the risk of recurrence remains high. However, the results of our study may resolve this problem by demonstrating that we can make tumor cells become vulnerable to chemotherapy by causing a reduction of dormant cells by stimulation with a growth factor. This may provide a novel therapeutic protocol in the treatment of colon cancer, while decreasing the potential risk for recurrence.
